# Development and validation of a prognostic nomogram for IgA nephropathy

**DOI:** 10.18632/oncotarget.21721

**Published:** 2017-10-10

**Authors:** Jian Liu, Shuwei Duan, Pu Chen, Guangyan Cai, Yong Wang, Li Tang, Shuwen Liu, Jianhui Zhou, Di Wu, Wanjun Shen, Xiangmei Chen, Jie Wu

**Affiliations:** ^1^ Department of Nephrology, Chinese People’s Liberation Army (PLA) General Hospital, Chinese PLA Institute of Nephrology, State Key Laboratory of Kidney Diseases, National Clinical Research Center for Kidney Diseases, Beijing Key Laboratory of Kidney Diseases, Beijing, China

**Keywords:** nomogram, IgA nephropathy, prognosis, pathology, risk

## Abstract

IgA nephropathy (IgAN) shows strong heterogeneity between individuals. IgAN prognosis is associated with pathological lesions and clinical indicators. However, simple tools for evaluating the clinical prognosis remain inadequate. Our objective was to develop an intuitive estimation tool for predicting the IgAN prognosis. 349 patients with IgAN at The Chinese People’s Liberation Army General Hospital were retrospectively analyzed from data between 2000 and 2006. A nomogram was developed using COX regression coefficients to predict decline of estimate Glomerular filtration rate (eGFR) ≥ 50% and end-stage renal disease (ESRD). The discriminative ability and predictive accuracy of the nomogram was determined via concordance index (C-index) and calibration curve. The results were verified in an independent validation cohort. In the derivation cohort, the nomogram was developed using mesangial hypercellularity, tubular atrophy/interstitial fibrosis, average proteinuria (A-P), and average mean arterial pressure (A-MAP) during hospitalization. The C-index of the nomogram was 0.88 (95% CI, 0.80 to 0.96). The calibration curve showed good agreement between prediction and actual observation. Furthermore, the nomogram demonstrated good discrimination (C-index = 0.87, 95% CI 0.78 to 0.95) and calibration in the validation cohort. The nomogram could predict the prognosis of IgAN effectively and intuitively.

## INTRODUCTION

IgA nephropathy (IgAN) is the most prevalent primary glomerulonephritis worldwide. IgAN is characterized by mesangial deposition of polymeric IgA1. Although IgA nephropathy progresses relatively slowly, the prognosis differs quite significantly among different individuals; therefore, a more refined prognostic evaluation has been the focus of researchers. The prognosis of IgA nephropathy is associated with risk factors [1] including pathological and clinical indicators. The Lee grade [2] and the Haas grade [3] are widely used pathological staging systems in clinics. In recent years, the Oxford classification [4] was added to the list, including mesangial hypercellularity (M), endocapillary proliferation (E), segmental sclerosis (S), tubular atrophy/interstitial fibrosis (T), and crescent [5] (C). However, the risk of end-stage renal disease (ESRD) cannot be easily and directly evaluated via these staging systems. For example, it remains uncertain whether a patient with Oxford M0T0S1E1C1 will progress to ESRD after five years. Thus, the Oxford classification is more suitable to analyze prognostic risk factors for a group of patients than calculating individual risks. However, some clinical indicators, such as proteinuria [6–9], hypertension [10], and estimated glomerular filtration rate (eGFR), have also prognostic value [11, 12]. The aim of this study was to establish a tool to predict the outcomes of IgAN using clinical and pathological indicators.

## RESULTS

### Baseline characteristics and endpoints of patients

604 participants were enrolled in this study. In the derivation cohort, 349 patients with IgA nephropathy met the inclusion criteria and were entered into this study. In the validation cohort, 255 patients met the inclusion criteria. Baseline data and endpoints are listed in Table 1. The median follow-up was conducted after 84 months and 76.8 months in the derivation and validation cohort, respectively. Endpoint events during the follow-up were 64 and 32 in the derivation and validation cohort, respectively. Four patients who died without endpoints were defined as right-censored data. The causes of death were myocardial infarction (n = 1), acute cerebral infarction (n = 1), gastrointestinal hemorrhage (n = 1), and unknown disease (n = 1). In the derivation cohort, the 5-, 8-, and 10-year renal survival rates were 95.0%, 76.1%, and 63.9%, respectively. In the validation cohort, the 5-, 8-, and 10-year renal survival rates were 94.8%, 75.2%, and 61.3%, respectively.

**Table 1:** Baseline characteristics of the patients in the derivation and validation cohorts

	Modeling (n = 349)	Validation (n = 255)	P value
Sex (M/F)	189/160	126/129	0.28
Age (year)	35.42 ± 9.53	35.42±10.10	0.99
eGFR(No. of patients)			0.15
>90 ml/min/1.73m^2^	213	170	
60-90ml/min/1.73m^2^	97	62	
30-60 ml/min/1.73m^2^	18	10	
<30 ml/min/1.73m^2^	21	13	
A-P(No. of patients)			0.32
<0.5g/24h	128	88	
0.5-1g/24h	108	107	
1-3.5g/24h	85	48	
>3.5g/24h	28	12	
A-MAP(mmHg)	91.71±11.91	93.14±11.46	0.09
Oxford classification(No. of patients)			
M (M0/M1)	237/112	159/96	0.17
E (E0/E1)	302/47	237/18	<0.01
S (S0/S1)	100/249	75/180	0.86
T (T0/T1/T2)	115/170/64	105/108/42	0.07
C (C0/C1/C2)	288/60/1	213/41/1	0.83
Application of glucocorticoids or immunosuppressive agents (No/Yes)	196/153	147/108	0.74
ARB or ACEI (No/Yes)	54/295	34/221	0.486
endpoint(No/Yes)	285/64	223/32	0.06

### Independent prognostic factors in the primary cohort

A univariate COX regression analysis was performed using pathological indicators (M/E/S/T/C), clinical indicators (sex, age, proteinuria, mean arterial pressure, and eGFR), and treatment indicators (use of, glucocorticoids or immunosuppressive agents, ACEI/ARB). All variables are listed in Table 2. The results showed that variables with P < 0.05 included M, S, T, average proteinuria during hospitalization (A-P), average mean arterial pressure (A-MAP), eGFR < 30 mL/min per 1.73 m^2^, and use of glucocorticoids or immunosuppressive agents. In the multivariate analysis with backward stepwise, two pathologic measures (M and T) and two clinical variables (A-P and A-MAP) were selected as independent risk factors for endpoints.

**Table 2:** Univariate and multivariate COX regression analysis

	Univariate				Multivariate^a^			
	patients	End points	HR	95.0% CI	P	HR	95.0% CI	P value
Sex								
women	160	28	1.00 (reference)			NS		
men	189	36	0.82	0.49-1.35	0.82			
Age			1.11	0.99-1.04	0.20	NS		
eGFR								
>90 ml/min/1.73m^2^	213	25	1.00 (reference)			NS		
60-90ml/min/1.73m^2^	97	21	1.71	0.72-4.10	0.21			
30-60 ml/min/1.73m^2^	18	6	3.08	0.88-10.84	0.04			
<30 ml/min/1.73m^2^	21	12	5.88	2.30-14.99	<0.01			
A-P								
<0.5g/24h	128	8	1.00 (reference)			1.00 (reference)		
0.5-1g/24h	108	18	2.23	1.70-2.91	0.05	1.49	1.08-2.04	0.01
1-3.5g/24h	85	29	4.95	2.23-10.79	<0.01	2.23	1.24-3.64	<0.01
>3.5g/24h	28	9	11.02	3.25-22.47	<0.01	3.32	1.96-5.01	<0.01
A-MAP			1.04	1.02-1.06	<0.01	1.03	1.01-1.05	<0.01
Oxford classification								
Mesangial hypercellularity								
M0	237	20	1.00 (reference)			1.00	(reference)	
M1	112	44	4.73	2.78-8.04	<0.01	1.80	1.00-3.29	0.05
Endocapillary hypercellularity								
E0	302	56	1.00 (reference)			NS		
E1	47	8	1.26	0.59-2.67	0.55			
Segmental glomerulosclerosis								
S0	100	7	1.00 (reference)			NS		
S1	249	57	3.368	1.54-7.40	<0.01			
interstitial tubular atrophy/interstitial fibrosis								
T0	115	8	1.00 (reference)			1.00(reference)		
T1	170	22	3.77	1.41-10.07	<0.01	3.818	1.37-10.65	0.01
T2	64	34	23.70	9.01-62.30	<0.01	16.30	5.75-46.20	<0.01
Cellular or fabrocellular Crescent								
C0	288	49	1.00 (reference)			NS		
C1+C2	61	15	1.88	1.05-3.37	0.03			
Use of GC or IS								
no	196	27	1.00 (reference)			NS		
yes	153	37	2.19	1.31-3.68	0.03			
Use of ARB or ACEI								
no	54	6	1.00 (reference)			NS		
yes	295	58	1.59	0.68-3.70	0.28			

HR, hazard ratio; CI, confidence interval; A-P, average proteinuria; A-MAP, average mean arterial pressure; GC or IS, glucocorticoids or immunosuppressive agents; NS, not selected.

^a^Variables were selected by using a Cox proportional hazard model and a stepwise backward method.

### Prognostic nomogram based on COX regression to predict renal survival

**Figure 1:**
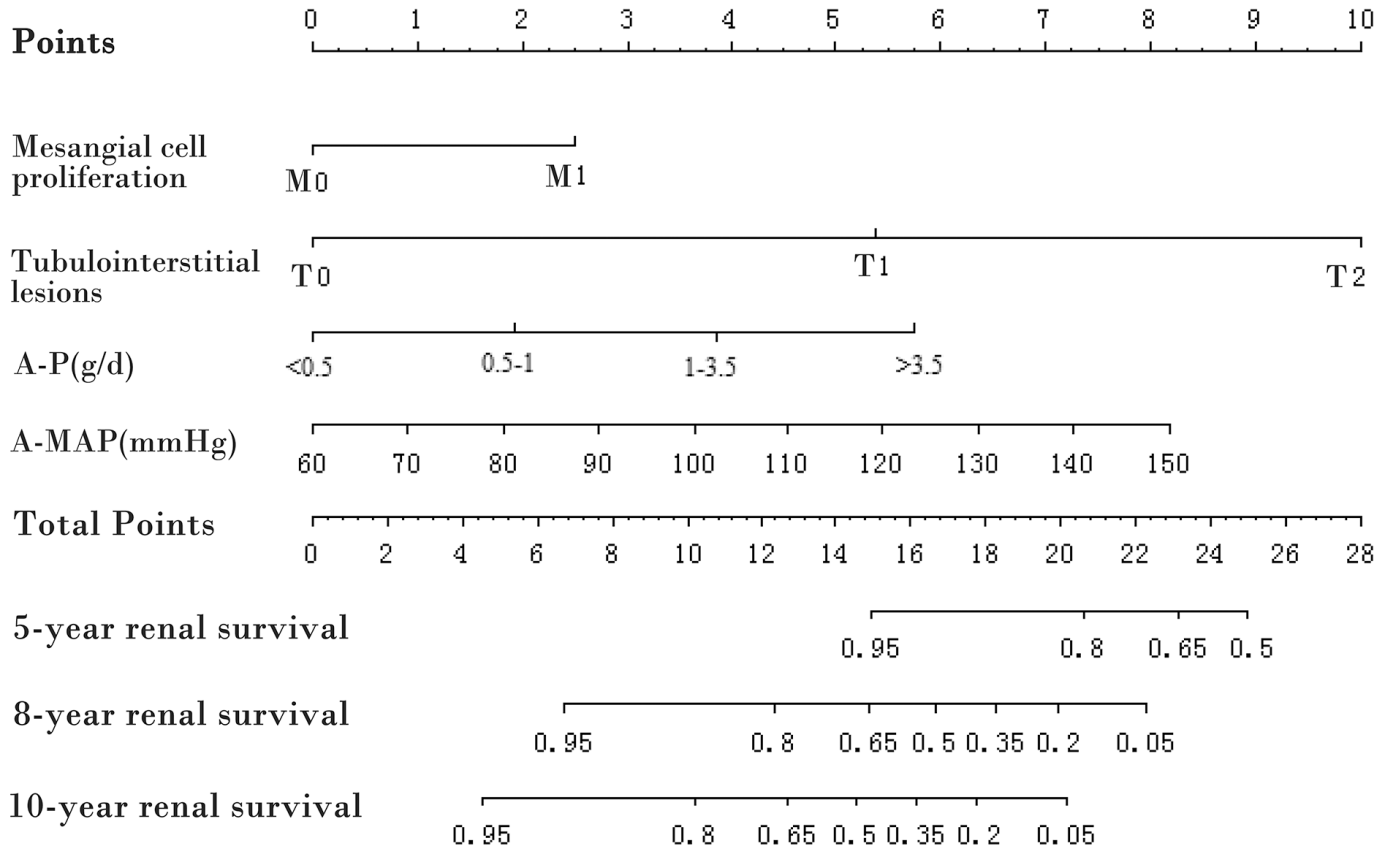
Prognostic nomogram A-P, average proteinuria; A-MAP, average mean arterial pressure. To use the nomogram, draw a line perpendicular from the corresponding axis of each risk factor until it reaches the top line labeled “Points.” Sum up the number of points for all risk factors then draw a line descending from the axis labeled “Total points” until it intercepts each of the survival axes to determine 5-, 8-, and 10-year survival probabilities.

**Figure 2:**
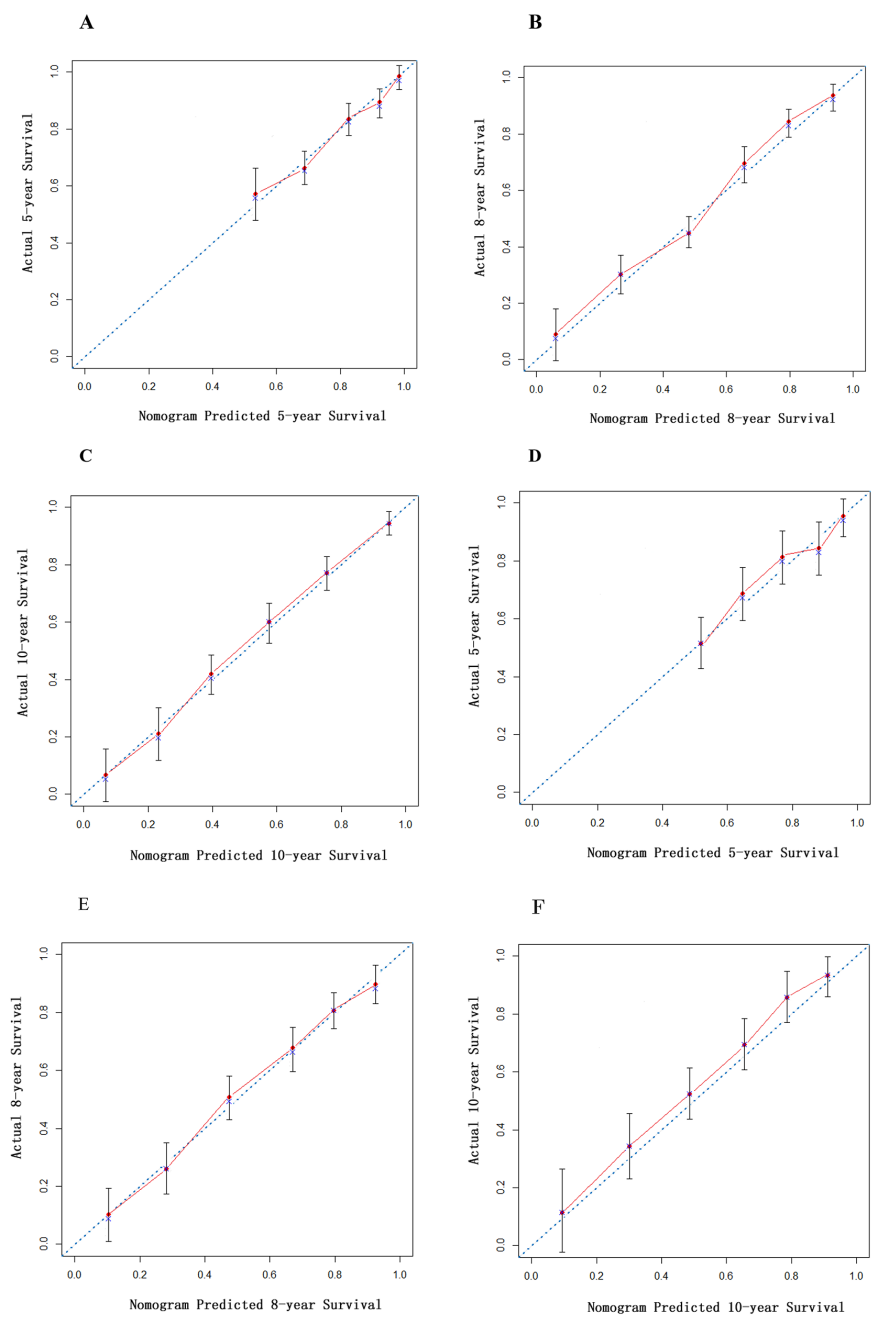
Calibration curve of nomogram in derivation cohort The calibration curve for predicting patient survival at **(A)** 5 years, **(B)** 8 years and **(C)** 10 years in the derivation cohort and **(D)** 5 years, **(E)** 8 years and **(F)** 10 years in the validation cohort. Nomogram-predicted probability of overall survival is plotted on the x-axis; actual overall survival is plotted on the y-axis.

A nomogram (Figure 1) to predict renal survival was developed using results from multivariate COX regression. Points were assigned to the four identified factors according to their regression coefficients. For each patient, the accumulated total points were compared to the survival scale to evaluate renal survival in different years. The concordance index (C-index) for the nomogram was 0.88 (95% confidence interval, 0.80 to 0.96). The calibration plot for the probability of survival at 5, 8, and 10 years after biopsy showed optimal agreement between the prediction via nomogram and the actual observation (Figure 2).

### Predictive accuracy of nomogram and other conventional classification systems in the derivation cohort

**Figure 3:**
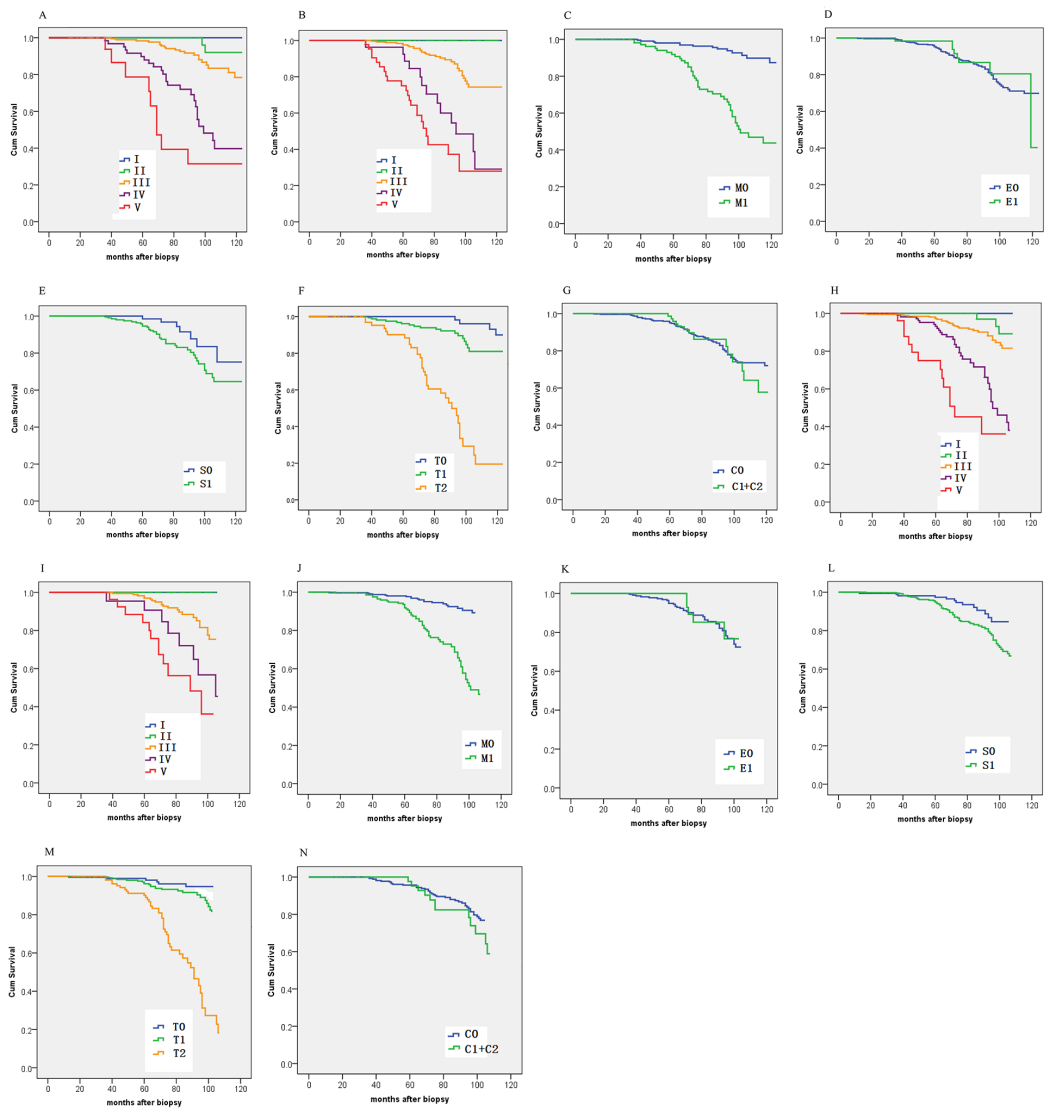
Kaplan-Meier survival curves of the derivation cohort (**[A]** Lee grade; **[B]** Haas grade; **[C]** Oxford M; **[D]** Oxford E; **[E]** Oxford S; **[F]** Oxford T; **[G]** Oxford C) and the validation cohort (**[H]** Lee grade; **[I]** Haas grade; **[J]** Oxford M; **[K]** Oxford E; **[L]** Oxford S; **[M]** Oxford T; **[N]** Oxford C) categorized by different staging systems.

As shown in Figure 3, Lee grade [2] and Haas grade [3] showed good prognostic stratification for patients between stage III and stage IV or later in both cohorts. However, in both cohorts, both Lee grade and Haas grade were unsatisfactory in stratifying patients between stages I and II. The mesangial hypercellularity (M0/M1), segmental sclerosis (S0/S1), and tubular atrophy/interstitial fibrosis (T0/T1/T2) of the Oxford classification showed good prognostic stratification for patients. However, crescent (C0/C1/C2) and endocapillary proliferation (E0/E1) were not as good as the other three indicators of the Oxford classification in distinguishing patients of both cohorts (Figure 3).

The C-index of the nomogram was 0.88. The calibration curve (Figure 2) of the nomogram showed good agreement between prediction and observation. These results suggest that our nomogram displayed good accuracy in predicting renal survival in the derivation cohort. In the same cohort, the C-index of the Lee grade, Haas grade, and Oxford Classification were 0.76, 0.74, and 0.78, respectively (Table 3). The nomogram seemed to have a higher C-index; however, the statistical difference between the nomogram and the other classification systems must be calculated in the validation cohort.

**Table 3:** C-index of the derivation cohort

Prognosis model	C-index (95% CI)
Nomogram	0.88(0.80-0.96)
Lee Grading	0.76(0.71-0.82)
Haas Grading	0.74(0.68-0.80)
Oxford classification	0.78(0.68-0.87)

### Comparison of predictive accuracy for renal survival between nomogram and other conventional classification systems in the validation cohort

In the validation cohort, the C-index of the nomogram was 0.87 (Table 4), which was higher than the Oxford classification (0.79), the Lee grade (0.77) [2], and the Hass grade [3] (0.74), (p < 0.01). The calibration curve (Figure 2) of the nomogram showed good agreement between prediction and observation in the probability of 5-year, 8-year, and 10-year renal survival.

**Table 4:** C-index of the validation cohort

Prognosis model	C-index (95% CI)
Nomogram	0.87(0.78-0.95)
Lee Grade	0.77(0.70-0.83) ^*^
Hass Grade	0.74(0.67-0.81) ^*^
Oxford classification	0.79(0.70-0.88) ^*^

^*^ p<0.01 compared with nomogram.

## DISCUSSION

In this study, we developed and validated a nomogram, consisting of four variables: A-proteinuria; A-MAP; Mesangial hypercellularity (M), and tubular atrophy/interstitial fibrosis (T). To select prognostic factors, Oxford M/E/S/T/C was not completely necessary after adjustment for clinical indicators. Other studies also support our result. Based on a Chinese population, Zeng et al. [31] conducted a multicenter study, enrolling 1,026 patients, and only M/T lesions were related to the prognosis of patients via multivariate analysis. Coppo et al. [32] reported a multicenter retrospective European-based VALIGA cohort study that comprised 1,147 IgAN patients. The results of their study were similar in so far that only the M/T lesions were found to be associated with the prognosis of patients using multivariate analysis. However, clinical indicators, such as baseline proteinuria, baseline blood pressure, and baseline eGFR were acknowledged as prognostic factors [33, 34]. However, clinical indicators always fluctuated and may be affected during a short time by treatment. Thus, we calculated the average mean arterial pressure and the average proteinuria during hospitalization instead of a one-time measurement, which enhanced the stability of these indicators.

Comparing the total points in the nomogram with the risk scale, we could comprehensively evaluate renal survival at different time points for each patient. The nomogram showed good discriminations for renal survival. The calibration curves exhibited a good fit with the observed incidence rate of renal survival in both the derivation and the validation cohorts for different years. Furthermore, the nomogram had a statistically improved accuracy in predicting renal survival compared to the pathological staging systems widely used in clinics such as the Lee grade, the Haas grade, and the Oxford classification. These results suggest that our nomogram is accurate for predicting long-term renal survival among Chinese IgAN patients.

In addition to commonly used pathological staging systems, some score systems to predict outcome of IgAN have been previously reported. However, these studies had several limitations. Berthoux et al. developed a risk score from data of 332 French patients [35], but their score evaluated histologic lesions using their own original pathologic classification system, which is not generally accepted. Xie et al. developed a risk score for predicting ESRD in 619 Chinese patients who were followed for an average of 3.4 years using eGFR, systolic pressure, serum albumin, and hemoglobin [36]. However, their score has not been verified in an independent validation cohort.

Several limitations of this study should be noted. First, the nomogram was established based on data obtained from a single institution in China. The generalizability for populations of other races and other countries may be limited. Second, patients with C2 according to the Oxford classification are too rare to have real statistical significance. The prognostic value of the crescent may be confirmed by research including more C2 patients. Third, patients with heavy proteinuria and advanced pathologic findings were likely to receive aggressive treatment. Both dosage and course of steroid and immunosuppressive agents is typically individualized. The effect of therapeutic intervention on the kidney prognosis needs to be confirmed by more clinical studies.

In conclusion, we have developed a nomogram that is scalable for the prognosis of IgA nephropathy and we verified its validity. This is a useful tool to estimate the individual risk for the outcome in patients with IgAN and for identifying those at high risk for future development of ESRD, which may be useful to determine the initial therapeutic strategies of patients with IgAN.

## MATERIALS AND METHODS

### Population

A retrospective study was conducted on a derivation cohort of patients who were diagnosed with IgAN according to a renal biopsy between January 2000 and December 2006 at The Chinese People’s Liberation Army General Hospital (Beijing, China). The inclusion criteria were as follows: 1) diagnosed as IgA via renal biopsy; 2) ≥ 18 years old; 3) eGFR ≥ 15 mL/min per 1.73 m^2^; and 4) number of glomeruli ≥ 8. The exclusion criteria were as follows: 1) secondary IgA nephropathy; 2) diabetes and other systemic diseases; 3) Follow-up < 36 months; 4) Patients who underwent several rounds of renal biopsy were not repeatedly enrolled. Another validation cohort was established to confirm the effectiveness of the nomogram. From January 2007 to December 2009, an independent cohort of consecutive patients with IgAN at the same institution formed the validation cohort of this study, using the same inclusion and exclusion criteria.

### Clinical measures

The sex, age, proteinuria, mean arterial pressure, and renal function were obtained from medical records. Mean arterial pressure (MAP) was defined as the diastolic pressure plus one third of the pulse pressure. An average of MAP (A-MAP) during hospitalization was calculated. Proteinuria was measured via 24-hour urine protein collection. In a similar manner to A-MAP, an average proteinuria (A-P) was calculated. Serum creatinine was measured with the enzymatic method. eGFR was calculated using the CKD-EPI formula for Asians [13]. The categorical classifications for urinary protein excretion were defined via traditional cutoffs of clinical significance [14].

### Pathological indicators

The biopsy specimens of the patients were stained and observed using hematoxylin and eosin, periodic acid–Schiff, periodic acid methenamine silver, and Masson’s Trichrome. Pathological changes were evaluated using the Oxford classification [5]. The mesangial hypercellularity score (M) was defined as M0 if the score was < 0.5 and as M1 if the score was > 0.5. Endocapillary hypercellularity (E) were expressed as E0 if absent and as E1 if present. Segmental glomerulosclerosis (S) was defined as S0 if absent and as S1 if present. Tuft adhesions were regarded as S1 lesions. Tubular atrophy/interstitial fibrosis (T) was semiquantitatively classified according to the percentage of the cortical area involved in the tubular atrophy/interstitial fibrosis: T0 for 0% to 25%, T1 for 26% to 50%, and T2 for > 50%. The crescent scores [15] (C) were defined as C0 (no cellular or fibrocellular crescents), as C1 (crescents in less than one fourth of all glomeruli), and C2 (crescents in more than one fourth of all glomeruli).

### Renal outcome

The study endpoint was determined as the progression to ESRD (and ESRD was defined as GFR < 15 ml/min per 1.73 m^2^) or a decrease in eGFR > 50% [16].

### Statistical analyses

Statistical analyses were performed using SPSS 22.0 (IBM Corporation, Armonk, NY, USA) and R programming software (version 3.3.3) [17]. A normal-distribution test using the Kolmogorov-Smirnov method was conducted on continuous variables and the results were expressed as the means ± standard deviation. Data with a non-normal distribution were expressed as the median. Count data were expressed as frequency. Normally distributed data were compared using a t-test; data that was not normally distributed and ranked data were compared using the rank sum test. Count data were compared with the chi-squared test. Multivariate analyses were performed using the COX proportional hazards model with backward stepwise [18, 19].

A nomogram [20, 21] is a graphical calculating device, a two-dimensional diagram, designed to allow for approximate graphical computation of a mathematical function. A nomogram was formulated based on the results of multivariate analysis and by using the package of rms in R (http://www.r-project.org/). Each factor was weighted via multivariate COX regression coefficients [22]. The renal survival was caculated by COX regression equation: h(t,x)=h0(t)exp(β1x1 + β2x2 +······+ βmxm).The predictive capacity of the model was evaluated using the concordance index (the Harrell C-Index) [23, 24] and assessed by comparing nomogram-predictions versus observed Kaplan-Meier estimates of survival probabilities [25–27]. Comparisons between the nomogram and other classification systems were performed via the C-index with the package of Hmisc in R [28]. A larger C-index resulted in a more accurate prognostic prediction [29, 30]. During the external validation of the nomogram, the total points of each patient in the validation cohort were calculated according to the established nomogram; then, Cox regression in this cohort was performed using the total points as a factor and finally, the C-index and calibration curve were derived based on the results of regression analysis.

### Ethical considerations

This study was conducted with the approval of the ethics committee of the PLA general hospital Review Board for Clinical Research. We have been approved to waive requirement for written, informed consent due to the retrospective nature of the study.

## Abbreviations

A-MAP: average mean arterial pressure during hospitalization
A-P: average proteinuria during hospitalization
C-index: concordance index
eGFR: estimate Glomerular filtration rate
ESRD: end-stage renal disease
IgAN: IgA nephropathy
